# Beyond non-inferiority: Exploring the full potential of virtual reality in surgical training

**DOI:** 10.1016/j.clinsp.2025.100675

**Published:** 2025-05-08

**Authors:** Brijesh Sathian, Hanadi Al Hamad, Javed Iqbal

**Affiliations:** a Rumailah Hospital, Hamad Medical Corporation, Doha, Qatar; b Nursing Department, Hamad Medical Corporation, Doha, Qatar

Dear Editor,

We are writing in response to the article titled *Virtual reality and traditional training in surgical instrumentation: A non-inferiority comparative study* published in your esteemed journal.^1^ While the study contributes valuable insights into the growing field of Virtual Reality (VR) in medical education, we have concerns regarding some of the claims made, particularly the assertion that VR is non-inferior to traditional surgical instrumentation training. However, we believe that this conclusion overlooks critical aspects of recent literature and requires further examination.

A major statement across the study illustrates that virtual reality training presents the advantages of safety combined with equal proficiency compared to conventional surgical instrumentation practices. This assessment lacks the breadth of evidence because VR demonstrates better results for teaching intricate surgical skills according to research studies in the field. VR demonstrates superior effectiveness for acquiring complex surgical skills than regular methods according to Lamb et al. (2023) as shown in their research.^2^ The study points to better outcomes for procedural memorization and skill execution at high-pressure conditions. The researchers did not evaluate how VR technology affects skills at more senior levels, so this present study did not demonstrate meaningful differences for basic instrumentation tasks. Research that declares VR training offers non-inferior outcomes fails to recognize its enhanced capabilities that become visible when high-fidelity simulations are incorporated.

The authors indicate that training with VR offers significant learning advantages because it creates immersive simulation environments that mimic operating room conditions. The combination of immersive virtual reality with increased motivation does not lead to superior procedural performance during surgeries compared to hands-on experiences in operational settings. Excessively using simulations with limited fidelity produces risks for surgical trainee overconfidence according to Massoth et al. (2019) because it impacts their performance during real interactions with actual patients.^3^ The study fails to discuss possible overconfidence among trainees who use the VR system although researchers should acknowledge whether this simulation tool properly prepares participants for unexpected events during actual surgical procedures.

Research data indicates that participants in the VR version demonstrate higher satisfaction levels compared to the traditional methods thus establishing that VR delivers higher engagement rates. The research of Stenseth et al. (2025) shows that simulated environments lead participants to report higher satisfaction levels especially if they are already experienced with technology.^4^ The authors should avoid using only subjectively measured scores to establish that VR training is better than traditional training because skill proficiency between groups remained equal.

The study lacks sufficient evidence to prove whether Virtual Reality training develops vital clinical skills that require critical thinking and decision-making skills under stressful conditions. Laspro et al. (2023) along with other recent studies demonstrate that VR training succeeds at skill development but fails frequently to involve students actively in important cognitive functions needed for genuine surgical practice.^5^ The reviewed study fails to evaluate essential surgical education aspects which guarantee that VR acts as more than a simple learning tool by supporting deep cognitive growth.

In conclusion, The study presents useful findings about VR surgical instrumentation education yet omits crucial aspects discovered in subsequent research about non-inferiority. Additional research needs to investigate VR's full effects on learning complex surgical abilities as well as cognitive mental focus and actual clinical practice effectiveness. Future medical education practices need deep insights into both the advantages and challenges of virtual reality technology to make effective decisions.

## Declaration of competing interest

The authors declare no conflicts of interest.
